# Retraction and replacement of: An integrated complete-genome sequencing and systems biology approach to predict antimicrobial resistance genes in the virulent bacterial strains of *Moraxella catarrhalis*

**DOI:** 10.1093/bfgp/elaf026

**Published:** 2026-02-02

This is a retraction and replacement of: Sadia Afrin Bristy, Md Arju Hossain, Md Imran Hasan, S M Hasan Mahmud, Mohammad Ali Moni, Md Habibur Rahman, An integrated complete-genome sequencing and systems biology approach to predict antimicrobial resistance genes in the virulent bacterial strains of *Moraxella catarrhalis*, *Briefings in Functional Genomics*, Volume 22, Issue 4, July 2023, Pages 375–391, https://doi.org/10.1093/bfgp/elad005

Following reader correspondence and an editorial assessment, Briefings in Functional Genomics is retracting the above article and replacing it with a corrected version.

In June 2025, a reader raised concerns about a set of issues in this article. The Editors determined that these issues, distributed across the description of results, assignment of mechanism and the breadth of claims, compromised the reliability and clarity of the published version and warranted retraction of the published version and publication of a corrected version. The Editors raised these concerns with the authors who acknowledged the issues and agreed to retract the original version of the article and replace it with a new version. In several places, the article conflated database derived AMR annotations and network centrality with mechanism and phenotypic resistance, which the authors now recognise as hypothesis generating and in need of experimental validation. Statements implying relevance to anti-tubercular agents (such as isoniazid and ethionamide) and the inclusion of triclosan were presented without sufficient context for *M. catarrhalis*; the revised text now distinguishes limits of the database annotations from clinical applicability. The article also misclassified several genes, including housekeeping “hub” genes, *fabG-1*, *ICR-Mc*, and *eptA/pgsA/OxyR*, as participating in specific resistance mechanisms (for example, target replacement or efflux) without supporting biochemical evidence; these assignments have been removed or corrected. A substantive factual error was identified in which *rpoB* mutations were associated with rifamycin susceptibility rather than resistance; this has been corrected. Finally, broad GO/KEGG functional enrichments (e.g., “cellular metabolic process,” “biosynthesis of secondary metabolites”) were discussed in a way that could be read as implying causation for antimicrobial resistance; the language has been revised to avoid inferring causation from correlation.

Edits span figure/text interpretation, mechanism assignments, statistical/terminology corrections, and statements on data scope and clinical relevance. The new version consolidates these changes. The new version has been reviewed and accepted by the editors and has been republished at https://doi.org/10.1093/bfgp/elaf027. The Authors apologise for errors in interpretation and presentation in the original version of the article.

